# Supplementary material for: Serum Neutralizing and Enhancing Effects on African Swine Fever Virus Infectivity in Adherent Pig PBMC
Source: Viruses. 2022 Jun 9;14(6):1249. doi: 10.3390/v14061249 (PMC9229155; doi:10.3390/v14061249)
Supplement: Supplementary file 1 [file viruses-14-01249-s001.zip › viruses-1727663-supplementary.pdf]

Supplementary Materials:

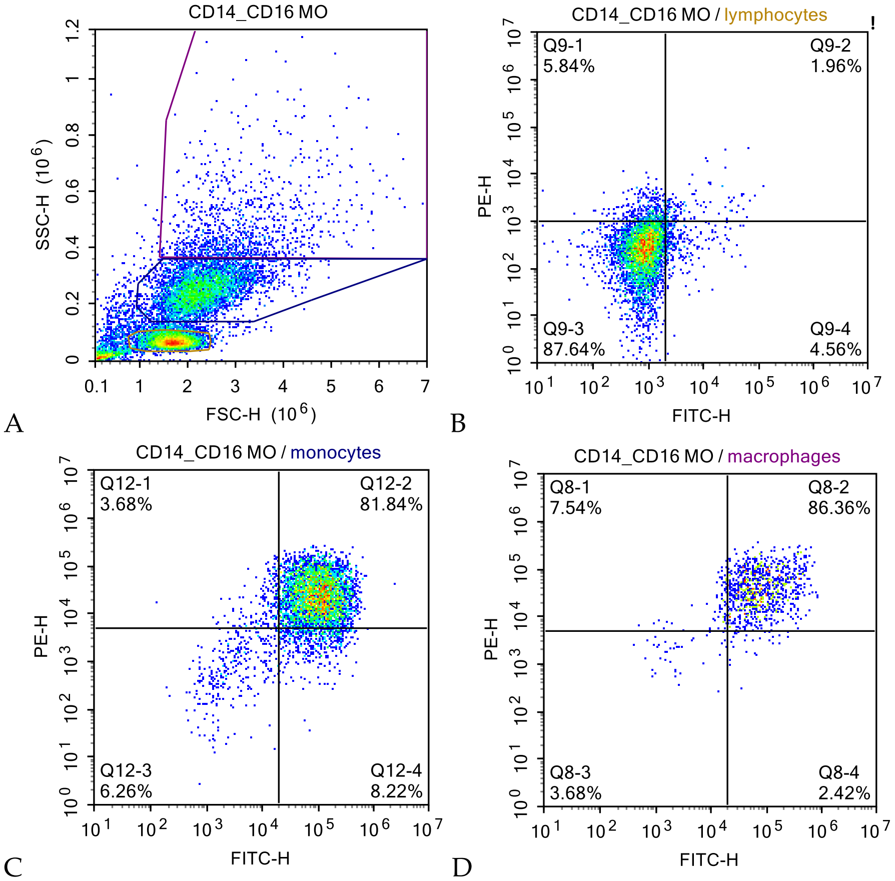

Supplemental Figure S1. Flow cytometry of non-infected adherent PBMC: (A) SSC-H vs FSC-H plot of adherent PBMC gated on lymphocytes, monocytes and macrophages; (B) CD14 (FITC) and CD16 (PE) staining of lymphocytes; (C) CD14 (FITC) and CD16 (PE) staining of monocytes; and (D) CD14 (FITC) and CD16 (PE) staining of macrophages

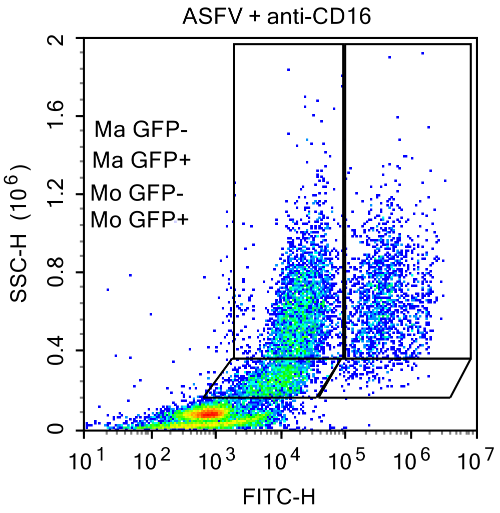

Supplemental Figure S2. The cell distribution on the SSC vs FITC (EGFP signal) plot of adherent PBMC gated on monocytes and macrophages after ASFV infection at MOI of 0.5 HAD_{50}

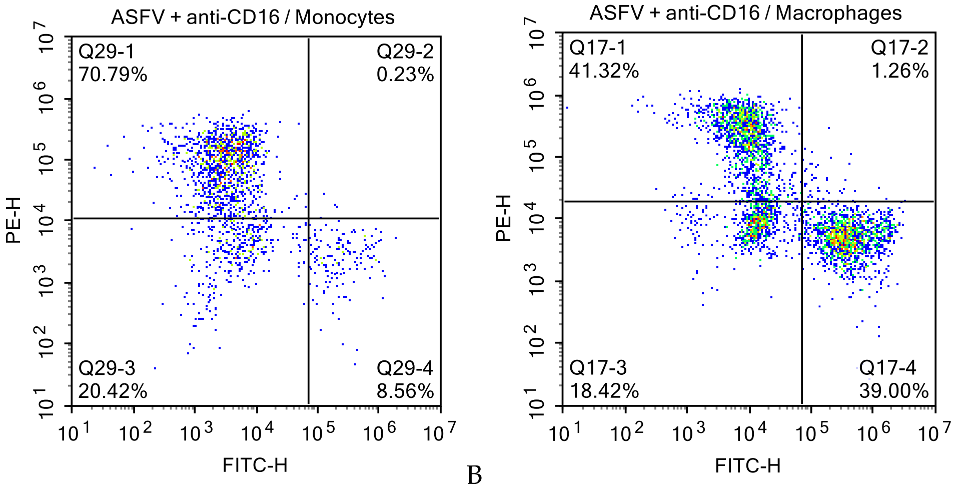

A B

Supplemental Figure S3. The EGFP (FITC) vs CD16 (PE) plots of ASFV infected adherent PBMC gated on (A) monocytes and (B) macrophages

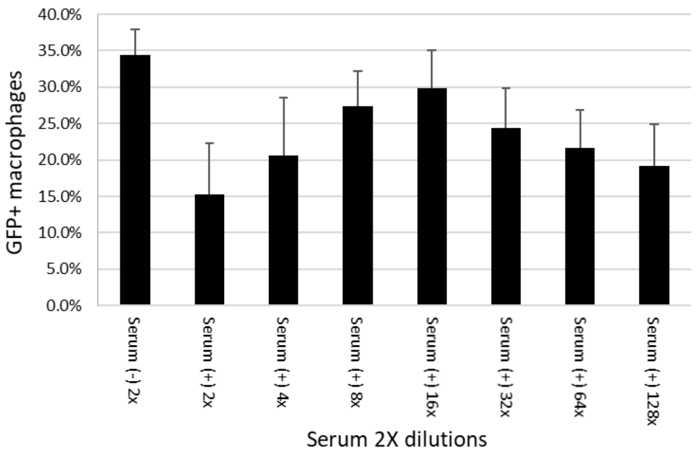

A

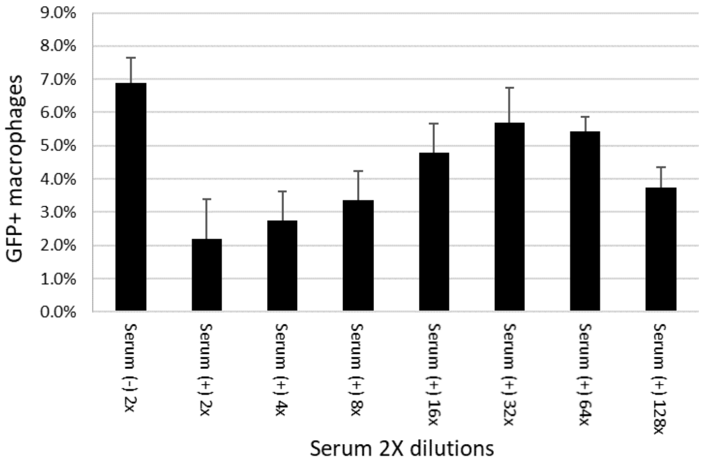

B

Supplemental Figure S4. The percentages of GFP+ macrophages treated with negative and hyperimmunized sera in 2X serial dilutions with culture medium as the diluent and ASFV infection at MOI of (A) 0.5 and (B) 0.05 HAD_{50} in adherent PBMC isolated from the same donor pig

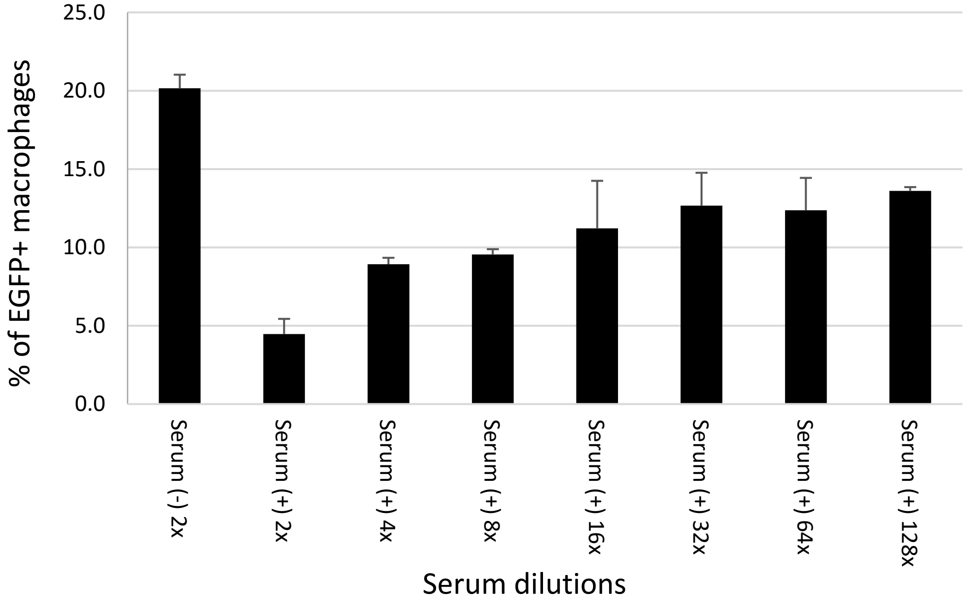

Supplemental Figure S5. The percentages of GFP+ macrophages treated with negative and hyperimmunized sera in 2X serial dilutions with a pooled negative serum as the diluents and ASFV infection at MOI of 0.5 HAD_{50} in adherent PBMC isolated from another donor pig
